# Ultra-Wide Band Radar Empowered Driver Drowsiness Detection with Convolutional Spatial Feature Engineering and Artificial Intelligence

**DOI:** 10.3390/s24123754

**Published:** 2024-06-09

**Authors:** Hafeez Ur Rehman Siddiqui, Ambreen Akmal, Muhammad Iqbal, Adil Ali Saleem, Muhammad Amjad Raza, Kainat Zafar, Aqsa Zaib, Sandra Dudley, Jon Arambarri, Ángel Kuc Castilla, Furqan Rustam

**Affiliations:** 1 Institute of Computer Science, Khwaja Fareed University of Engineering and Information Technology, Abu Dhabi Road, Rahim Yar Khan 64200, Punjab, Pakistan; ambreenakmal888@gmail.com (A.A.); adilalisaleem@gmail.com (A.A.S.); ch.amjadraza@gmail.com (M.A.R.); kainat.zafar96@gmail.com (K.Z.); aqsaz2613@gmail.com (A.Z.); 2 Institute of Computer and Software Engineering, Khwaja Fareed University of Engineering and Information Technology, Rahim Yar Khan 64200, Punjab, Pakistan; muhammad.iqbal@kfueit.edu.pk; 3 Bioengineering Research Centre, School of Engineering, London South Bank University, 103 Borough Road, London SE1 0AA, UK; dudleyms@lsbu.ac.uk; 4 Universidade Internacional do Cuanza, Cuito EN250, Angola; jon.arambarri@uneatlantico.es (J.A.); angel.kuc@unini.edu.mx (Á.K.C.); 5 Fundación Universitaria Internacional de Colombia, Bogotá 111321, Colombia; 6 Universidad Internacional Iberoamericana, Campeche 24560, Mexico; 7 Universidad de La Romana, La Romana 22000, Dominican Republic; 8 Universidad Europea del Atlántico, Isabel Torres 21, 39011 Santander, Spain; 9 School of Computing, National College of Ireland, Dublin D01 K6W2, Ireland

**Keywords:** drowsiness, ultra-wideband radar, convolutional neural network, spatial features, ensemble models

## Abstract

Driving while drowsy poses significant risks, including reduced cognitive function and the potential for accidents, which can lead to severe consequences such as trauma, economic losses, injuries, or death. The use of artificial intelligence can enable effective detection of driver drowsiness, helping to prevent accidents and enhance driver performance. This research aims to address the crucial need for real-time and accurate drowsiness detection to mitigate the impact of fatigue-related accidents. Leveraging ultra-wideband radar data collected over five minutes, the dataset was segmented into one-minute chunks and transformed into grayscale images. Spatial features are retrieved from the images using a two-dimensional Convolutional Neural Network. Following that, these features were used to train and test multiple machine learning classifiers. The ensemble classifier RF-XGB-SVM, which combines Random Forest, XGBoost, and Support Vector Machine using a hard voting criterion, performed admirably with an accuracy of 96.6%. Additionally, the proposed approach was validated with a robust k-fold score of 97% and a standard deviation of 0.018, demonstrating significant results. The dataset is augmented using Generative Adversarial Networks, resulting in improved accuracies for all models. Among them, the RF-XGB-SVM model outperformed the rest with an accuracy score of 99.58%.

## 1. Introduction

Drowsiness manifested by drooping eyes, mind wandering, eye rubbing, inability to concentrate, and yawning, is a state of fatigue that presents a substantial danger, especially when it comes to road safety. Recent investigations highlight the seriousness of the problem, revealing that 30% of the 1 million deaths caused by road accidents can be related to driver weariness or drowsiness [1,2]. The likelihood of a collision increases thrice when the driver is experiencing weariness, emphasizing the importance of taking preventative steps. The American Automobile Association (AAA) has discovered that there are approximately 328,000 crashes caused by drowsy driving each year [3]. These crashes have had a significant impact on society, costing almost 109 billion USD, not including property damage [3]. This staggering figure encompasses immediate and long-term medical expenses, productivity losses in both workplace and household contexts, legal and court costs, insurance administration expenses, and the economic impact of travel delays. Specific demographic groups are particularly susceptible to drowsiness while driving. Night-shift male workers and individuals with sleep apnea syndrome emerge as high-risk categories [4]. Several research studies have been published, suggesting strategies to mitigate or notify drivers about possible indications of drowsiness [5,6,7,8,9,10,11,12,13,14]. These measures are important steps in tackling the critical issue of drowsy driving and improving road safety.

Drowsiness detection systems can be classified into three main categories: vehicle dynamics, physiological signals, and recognition of driver face characteristics [11,12,15,16]. Nevertheless, the efficacy of vehicle dynamics-based systems is hindered by the suboptimal performance caused by unpredictable variables such as road geometry, sluggish processing speed, traffic conditions, and head movement [15,16,17]. Conversely, the examination of yawning and blinking by analyzing facial images of the driver has shown potential in controlled or virtual environments [16,17]. However, the performance of these systems often decreases when used in real-world settings due to factors including changes in lighting, differences in skin color, and temperature fluctuations [16,17]. Conversely, systems relying on physiological signals have demonstrated a high level of accuracy, establishing them as a dependable approach for real-world applications. Physiological measures such as electroencephalography (EEG) [6,18,19,20,21,22,23,24], electrooculography (EOG) [25,26,27,28,29,30], respiration rate [12,31,32,33,34,35], electrocardiography (ECG) [34,36,37,38], and electromyography (EMG) [39,40,41,42,43] are commonly used in the systems designed to identify driver drowsiness. Although the sensors used to capture these signals are effective, a significant obstacle occurs due to their invasive character, making it difficult to integrate or practically use them in real-world contexts.

Among these physiological signals, the respiration rate is especially noteworthy because it fluctuates significantly from awake to sleep and varies depending on numerous physiological situations. In addition, the respiratory system undergoes modifications during sleep, which are impacted by decreased muscle tone and shifts in chemical and non-chemical reactions [44]. It is worth mentioning that a decline in the rate at which a person breathes is frequently noticed prior to a driver reaching a state of sleep [45,46]. This study aims to address the challenge of accurately detecting driver drowsiness in real time using UWB radar signals and advanced machine learning (ML) techniques. The primary objectives are to develop robust feature extraction methods, design efficient ensemble models, and validate their effectiveness against existing methods. In this manuscript, the proposed system employs the non-invasive acquisition of chest movement through Ultra-Wideband (UWB) radar to distinguish between the drowsy and non-drowsy states of the driver. UWB radar offers notable benefits such as fast data rates and low power transmission levels [47]. This is achieved by transmitting very short-duration pulses, resulting in signals with wide bandwidth. The technology does not raise any privacy problems because it is not influenced by ambient elements, does not rely on light or skin color, and emits very little power, guaranteeing human safety [48,49,50]. Furthermore, the system maintains its resilience even when exposed to Wi-Fi and mobile phone transmissions. The UWB radar’s ability to penetrate different materials or obstructions, combined with its non-intrusive nature [51,52], makes it an excellent option for this drowsiness detection system. The chest readings obtained are subsequently transformed into grayscale images, as illustrated in [53], and these images are utilized as input deep learning (DL) models. The features extracted from these models are then employed to train and test ML algorithms. The contributions of this study are as follows:

- This system utilized a dataset from [12] and transformed it into grayscale images for analysis.
- The system employs Convolutional Neural Network (CNN) architecture to extract features from these images.
- These features are input into various machine learning (ML) algorithms, and the performance of these algorithms is assessed on a test set.
- The hybrid ensemble models RF-MLP and RF-XGB-SVM have been developed to combine the unique capabilities of multiple algorithms.
- The models undergo evaluation using metrics such as accuracy, precision, recall, and F1 score. In the end, a comparative analysis is conducted to determine which deep learning-based feature yields superior results.

This paper is organized into several sections. Section 2 presents the literature review of the study, while Section 3 describes the methodology of the proposed approach. Section 4 presents the results, and finally, Section 5 contains the study’s conclusion.

## 2. Literature Review

The literature review examines various prominent studies that specifically investigate the identification and categorization of drowsy and alert conditions in drivers. The classification of drowsy and non-drowsy states is accomplished by utilizing non-invasive IR-UWB radar to measure the breathing rate, as stated in the research [12]. The chest motions of 40 individuals were collected, and the Support Vector Machine algorithm achieved an accuracy rate of 87%. The study demonstrates the efficacy of UWB in detecting driver drowsiness by analyzing breathing rates. The paper introduces an EEG-based spatial-temporal CNN (ESTCNN) in [54] to detect driver fatigue. The network independently acquires characteristics from EEG inputs, with an exceptional classification accuracy of 97.37%. The experiments involve the collection of EEG signals from eight participants in both alert and fatigue stages. The research presented by [55] focuses on two distinct categories of videos: alert and drowsy. The study utilizes a thorough dataset consisting of 60 individuals who have been classified into three groups: alert, low vigilant, and drowsy. Two separate models are created, utilizing computer vision and deep learning to analyze temporal and spatial features. Ref. [56] suggests a method of evaluating exhaustion that does not require any intrusive procedures. This method involves analyzing physiological signs such as heart rate variability (HRV) and ECG data. During sleep periods, ECG data are collected, and the continuous wavelet transform is used to extract features. The average accuracy achieved via ensemble logistic regression is 92.5%, with a processing time of 21 s. Ref. [57] improves the detection of drowsiness by combining ECG and EEG features. The data collected from 22 participants in a driving simulator exhibit noteworthy characteristics that differentiate between states of being alert and tired. By combining modalities, Support Vector Machine (SVM) classification produces enhanced performance, while channel reduction ensures accuracy using only two electrodes.

The Intelligent Drowsiness Detection System (DDS) described in [58] uses Deep Convolutional Neural Networks (DCNNs), specifically VGG16, InceptionV3, and Xception, to address driver fatigue. The Xception model has exceptional performance, with an accuracy of 93.6%. It surpasses both the VGG16 and InceptionV3 models when applied to a dataset containing facial recordings depicting drowsy and non-drowsy states. In [59], an approach with two phases tackles the difficulties in intelligent transportation systems by presenting an improved fatigue detection system that relies on DenseNet. The system consists of a module that represents the model and a sophisticated method for channel attention. The second stage utilizes a guided policy search (GPS) algorithm to facilitate collaborative decision-making, adjusting to the current levels of driver fatigue in real time. Empirical validation on datasets such as YaWDD, RLDD, and DROZY showcases substantial enhancements and achieved an average accuracy of 89.62%. The fatigue detection method, implemented in [60], utilizes powerful CNN models to specifically target yawning. This system demonstrates a remarkable accuracy of 96.69% on the YaWDD dataset. The analysis demonstrates that data augmentation achieves a trade-off between accuracy and model resilience, resulting in a modest decrease in accuracy but an improvement in the model’s ability to withstand complications. In [61], a novel deep learning approach for driver drowsiness identification utilizes a MobileNet-SSD CNN with the SSD technique. Trained on a diverse dataset of 6000 photos, the model achieves a substantial Mean Average Precision (mAP) of 0.84, prioritizing computing efficiency for real-time processing on mobile devices. The methodology incorporates a unique dataset from various sources, ensuring diverse representation. Experimental results demonstrate the model’s resilience, achieving high mAP values for closed eyes (0.776), open eyes (0.763), and outstanding face detection (0.971).

In a study conducted by researchers [62], a Regularized Extreme Learning Machine (RELM) showed exceptional performance in identifying driver drowsiness. The RELM achieved an accuracy rate of 99% using a dataset consisting of 4500 pictures. The combination of video surveillance, image processing, and ML [63] results in a sleepiness detection system that achieves a 93% accuracy rate. This accuracy is determined by analyzing eye blink patterns from the YawDD dataset. The system described in [64] utilizes the PERCLOS algorithm, Python modules, and ML techniques to evaluate eye movements. It achieves a high accuracy rate of 93% in the real-time detection of driver drowsiness. The utilization of mmWave FMCW radar enables [65] to reach an accuracy of 82.9% in detecting drowsiness. This is accomplished by collecting chest motions and employing ML methods. Ref. [66] integrates MTCNN facial detection with GSR sensor-based physiological data, resulting in an accuracy of 91% in the real-time detection of driver drowsiness. The study [67] combines behavioral metrics and physiological data, utilizing Raspberry Pi and SVM classifiers, to achieve a commendable accuracy rate of 91% in detecting driver tiredness. The study [68] uses a histogram of oriented gradients (HOG) and linear SVM to achieve outstanding precision. The DDS in [69] uses CNN to extract features, resulting in an accuracy rate of 86.05% on a dataset of 48,000 photographs. The study [70] conducted in Zimbabwe mainly addresses the issue of road safety. It successfully achieves a detection accuracy of over 95% in identifying drowsiness. This is accomplished by the implementation of the principal component analysis (PCA) dimensionality reduction technique, along with classifiers such as XGBoost and Linear Discriminant Analysis. The implementation of a real-time drowsiness detection system on Nvidia Jetson Nano, as described in [71], achieves an accuracy rate of 94.05%. In particular, it excels particularly in detecting yawning. The paper [72] presents a DDS that uses webcam-based surveillance to detect drowsiness in real-time. The system achieves a high level of accuracy, with over 97% accuracy in multiple metrics such as precision, sensitivity, and F1-score. Ref. [55] presents a DDS that operates in real-time. The system utilizes the Viola–Jones algorithm, a beeping sound mechanism, and calculates the distance between the lips. This combination of technologies provides scalability and cost-effectiveness, which ultimately leads to increased road safety.

Although these investigations contribute substantially to the field of driver drowsiness detection, it is important to highlight numerous limitations. Although video-based methods are successful in controlled environments, they can face difficulties in real-world situations due to inconsistent lighting conditions, which could potentially affect the precision of drowsiness detection. Furthermore, the adoption of physiological data-centric systems in real-time is hindered by practical problems arising from the invasive nature of on-body sensors, regardless of their effectiveness. Not only does this give rise to privacy problems, but it also obstructs the smooth incorporation of such technologies into ordinary driving situations. Hence, the implementation of these techniques in actual driving scenarios requires careful deliberation of these limitations.

## 3. Methodology

**Figure 1:**
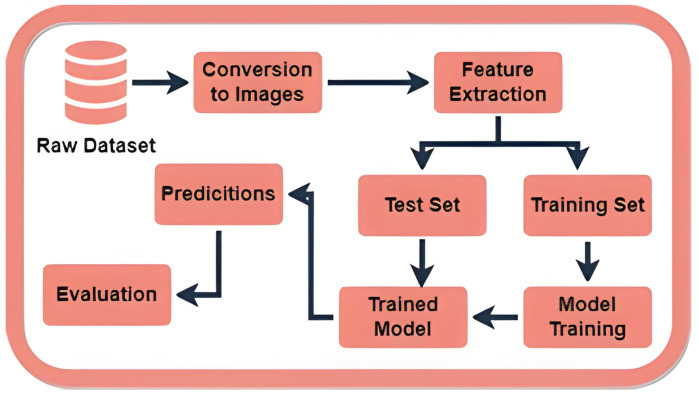
Proposed methodology diagram of the system.

The proposed methodology is depicted in Figure 1. Initially, a data set was sourced from [12], which included chest movement signals acquired through the UWB radar in both drowsy and non-drowsy states. Subsequently, the dataset was transformed into grayscale images, which were then used as input for a CNN model. The Features were extracted from the images using the DL model and then saved in a CSV file, along with their accompanying labels. Following that, the dataset was divided into two distinct sets: the training set and the testing set. The training set was utilized to train various ML classifiers, whilst the test set was put aside for evaluating the performance of these models. The test set was used to make predictions, which were then evaluated using key metrics like accuracy, precision, recall, and F1 score.

### 3.1. Dataset

**Figure 2:**
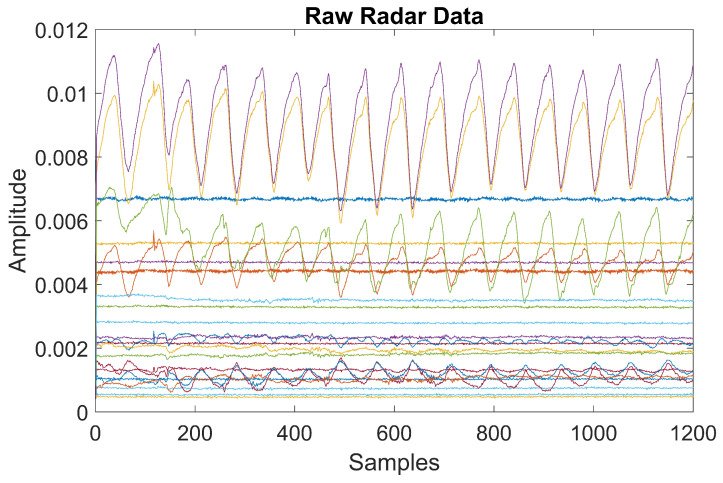
Raw radar signal of chest movement.

The dataset used in this investigation was obtained from [12] which comprises the chest movements of the drivers in the drowsy and non-drowsy state using the X4M300 UWB radar (NOVELDA, Oslo, Norway). The experiment involved forty professional male drivers engaged in extended intercity driving sessions that lasted approximately 10 h. For non-drowsy states, chest movement data were recorded before the drivers commenced their driving shifts. Conversely, in the case of fatigued data, the chest data of the same participants were collected shortly after they finished a 10-h driving shift. The raw radar signal of the chest movement is shown in Figure 2.

**Figure 3:**
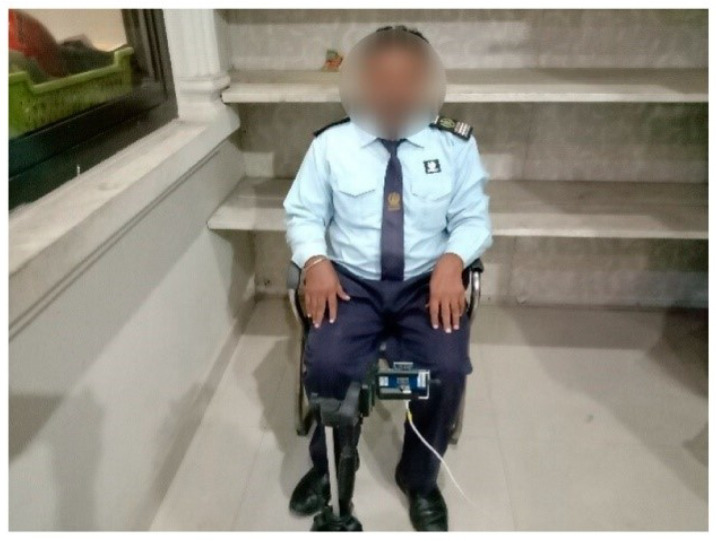
Subject in front of the radar while collecting data obtained from [12].

In order to promptly evaluate the drivers following their return from their trips, a specially designed testing area was established in a vacant room at the Manthar Transport Company’s terminal in Sadiq Abad, Punjab, Pakistan. Throughout the process of collecting data, drivers were given instructions to position themselves directly in front of the radar. A minimum distance of 1 m was maintained between the radar and the subject at chest level, as shown in Figure 3. The radar had a range of 9.4 m from the transmitter-receiver point, allowing it to detect any movements within this distance. The selected 1-meter distance was predicated on the supposition that the driver could be in any position within this range while operating the vehicle. The radar device was positioned at chest level to guarantee that the subject stayed inside the radar’s effective range. Practically, the distance from the dashboard to the human body could range from 0.2 m to 0.5 m. The chest movement from each subject was collected for five minutes and stored in a CSV file.

### 3.2. Conversion to Images

Each file consists of a five-minute recording of the chest movement, which is then divided into one-minute segments and labeled as matrix “A”. An approach is utilized to produce grayscale image representations of the matrix “A”, which are denoted as “I”. The purpose of this conversion is to visually depict the matrix, hence improving the comprehensibility and analysis of the data. In order to accomplish this, the ’mat2gray’ function in MATLAB R2020a is utilized. The function initially identifies the minimum and maximum values in the input matrix. Using these values, a normalization formula as shown in Equation (1) is applied to each element, subtracting the minimum value and then dividing by the difference between the maximum and minimum values. This procedure adjusts the minimum value of the matrix to 0 and rescales the maximum value to 1, so ensuring that all other values proportionally fall within this range. If the minimum and maximum values of a matrix are equal, which implies that all values in the matrix are the same, mat2gray assigns a value of 0.5 to all output values. This is executed to prevent undefined operations and provide a reasonable default value.

(1) $$I=\frac{A-\min\left(A\right)}{\max\left(A\right)-\min\left(A\right)}$$

**Figure 4:**
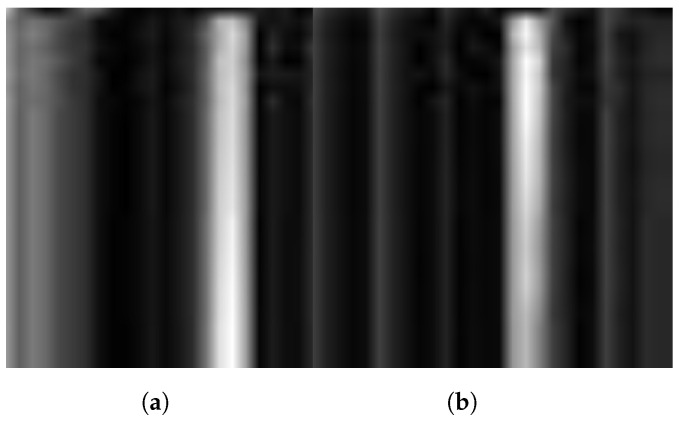
Converted grayscale images of (**a**) drowsy class (**b**) fresh class.

Here, ‘min(A)’ represents the minimum value within matrix ‘A’, and ‘max(A)’ signifies the maximum value within the same matrix. The procedure entails subtracting the minimum value from each element of ‘A’ and then dividing the resulting value by the range of values, which is the difference between the maximum and minimum values. The normalization technique guarantees that the values in the matrix ‘A’ are rescaled to fit inside the typical grayscale image representation range of [0, 1]. The converted images for both drowsy and fresh classes are shown in Figure 4.

### 3.3. Feature Extraction

**Figure 5:**
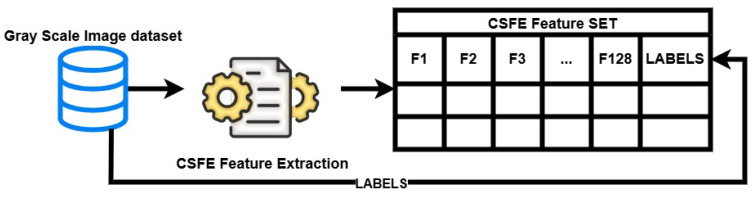
The architecture diagram of CSFE feature extraction.

The study utilized the Convolutional Spatial Feature Engineering (CSFE) method, as presented in [53], to extract spatial features from grayscale images. The process is visualized in Figure 5. By incorporating 2D convolutional layers into CNN architectures, this technique enables the extraction of complex spatial features from the image data. The spatial features obtained provide a thorough depiction, encompassing intricate patterns and movements that are essential for a range of applications. In this research, CSFE features were derived from grayscale images, forming a new feature set along with corresponding labels. These features are then used to train and evaluate ML models, with the goal of accurately detecting driver drowsiness. By harnessing the spatial information extracted through CSFE, these models exhibit the potential to discern nuanced patterns and movements often overlooked by conventional CNNs, thereby enhancing accuracy in drowsiness detection. The architecture of the 2D CNN used in this research is given in Table 1. The rescaling layer performs an initial normalization of the pixel values, guaranteeing a uniform input range of [0, 1]. The initial convolutional layer has 64 filters and a 3 × 3 kernel to identify basic patterns and edges in the input image. It utilizes the ReLU activation function to introduce non-linearity and improve the representation of features. Following max pooling decreases spatial dimensions, preserving important characteristics while decreasing computational complexity.

The second convolutional layer utilizes 32 filters to enhance the process of extracting features while using additional max-pooling to assist in reducing spatial dimensions. The flattened layer prepares the data for fully connected layers, facilitating global feature integration. The 128-neuron dense layer refines the hierarchical features in order to capture intricate patterns and relationships. This architecture is a result of the run-and-test method, demonstrating its adaptability to the specific characteristics of the data set. The selection of these layers aims to achieve an in-depth balance, allowing for efficient feature extraction without introducing unnecessary complexity. The architecture’s advantages stem from its capacity to systematically extract complex spatial characteristics from images. These 128 features are stored in a CSV file along with labels for the classification of the drowsy and non-drowsy states of the drivers.

### 3.4. Data Augmentation

**Figure 6:**
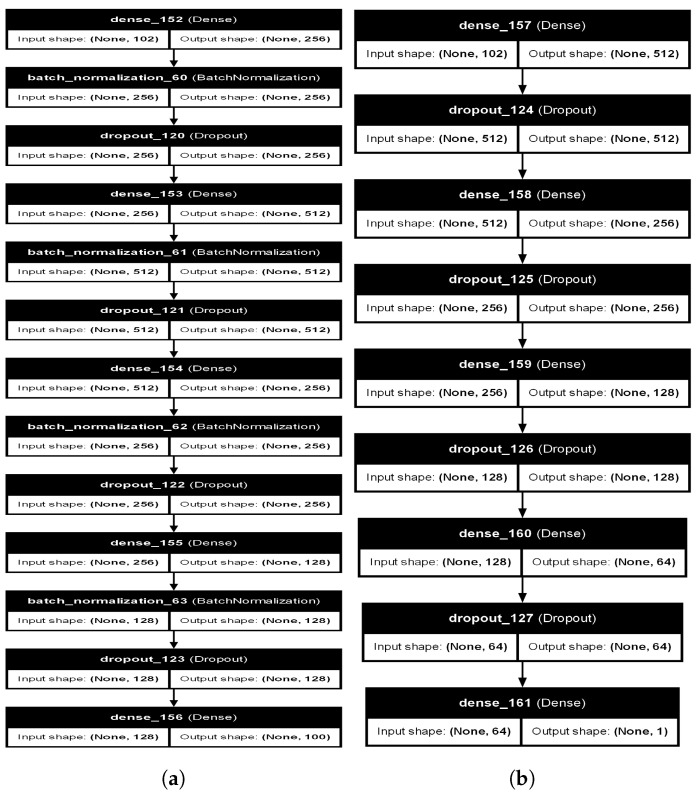
The architecture of GAN (**a**) Generator (**b**) Discriminator.

The study presented in this manuscript employs Generative Adversarial Networks (GANs) to address the problem of a small dataset size. Specifically, the dataset used in this manuscript contains only 200 instances per class, which is insufficient for training robust ML models. GANs, which were proposed by Ian Goodfellow [73], are a type of ML framework specifically created to produce artificial data that closely resemble a given dataset. A GAN comprises two neural networks: a Generator and a Discriminator. The Generator produces novel, artificial data instances, while the Discriminator assesses them to differentiate between genuine and artificial (counterfeit) data. The two networks are trained concurrently in a competitive environment: the Generator grows its proficiency in generating actual data, while the Discriminator improves its ability to identify counterfeit data. The Generator model is specifically designed to accept a random noise vector as input and convert it into a synthetic data instance that closely mimics the actual data. The structure of the Generator commences with an input layer that receives a noise vector of 102 dimensions. Subsequently, a sequence of compact layers is employed with the objective of gradually enhancing the data representation. The initial dense layer is composed of 256 neurons that utilize the Rectified Linear Unit (ReLU) activation function. This is then followed by batch normalization and a dropout layer with a 30% probability. These measures are implemented to enhance stability and mitigate overfitting. The second dense layer consists of 512 neurons, which are likewise activated using the ReLU function. Additionally, batch normalization and dropout layers are applied. The architecture then incorporates a third layer that is densely populated with 256 neurons, and a fourth layer with 128 neurons. Both layers adhere to the identical sequence of activation, normalization, and dropout. The Generator’s final output layer generates a 100-dimensional vector via linear activation, which represents the synthetic data that have been created. The architecture of the generator is shown in Figure 6a.

The Discriminator model’s objective is to distinguish between genuine data from the dataset and the artificial data produced by the Generator. The process starts with an input layer that receives a data vector consisting of 102 dimensions. The first layer of the Discriminator consists of 512 neurons with ReLU activation, which is then followed by a dropout layer with a 30% probability in order to mitigate overfitting. Following this, there are further layers with a high concentration of neurons, namely 256, 128, and 64 neurons, respectively. Each of these layers is activated using the ReLU function and includes dropout layers. The final output layer of the Discriminator is a single neuron with sigmoid activation, outputting a probability score that indicates whether the input data are real or synthetic. The architecture of the discriminator model is shown in Figure 6b.

During the training phase, the Generator and Discriminator participate in a two-player minimax game. The Discriminator is trained by being presented with batches of real data and data produced by the Generator. It learns to improve its ability to distinguish real data from fake data. Simultaneously, the Generator is trained to generate artificial data that can deceive the Discriminator into categorizing it as authentic. The process of adversarial training persists until the Generator generates data that are indistinguishable from genuine data, therefore, substantially enhancing the original dataset with synthetic examples of high quality. Using GANs, the dataset is effectively augmented by adding 1000 instances to each class, resulting in a total of 1200 instances for each class. This enhancement facilitates the creation of more robust and accurate ML models. The sample of the augmented data is shown in Table 2.

### 3.5. Proposed Ensemble Models

**Figure 7:**
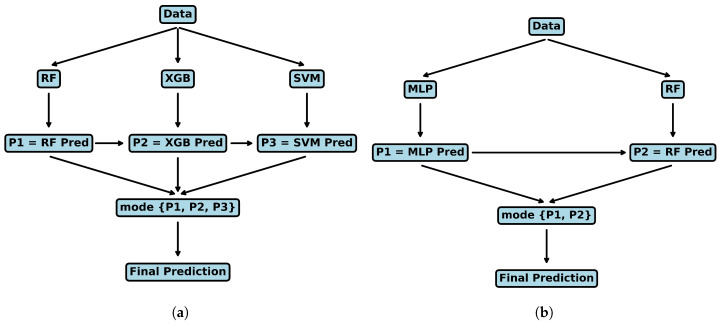
The architecture of ensemble models (**a**) RF-XGB-SVM, (**b**) RF-MLP.

In this manuscript, in addition to individual ML models, two ensemble models RF-MLP and RF-XGB-SVM are proposed with hard voting for the classification task between drowsy and fresh classes. The rationale behind the selection of the RF-MLP and RF-XGB-SVM models is to exploit the advantages of various methods in order to improve the accuracy of predictions. RF-MLP is a hybrid model that combines the robustness of RF with the deep learning skills of MLP. On the other hand, RF-XGB-SVM is a model that merges the strong boosting powers of Extreme Gradient Boosting with the effectiveness in handling high-dimensional data of SVM. The voting mechanism among these separate ensembles introduces diversity, robustness, and computational efficiency, allowing us to balance accuracy and model interpretability. The architecture of both ensemble models is presented in Figure 3. Here, in Figure 7, P1, P2, and P3 the predictions of the respective classifiers, and in the final classification, the class with the majority of votes among the predictions will be selected as the final prediction.

The Algorithm 1 outlines the procedural steps employed by the RF-MLP ensemble model following the hard voting criteria. The trained Random Forest (TRF) and Trained Multilayer Perceptron (TMLP) models operate on the feature vector to predict whether a given sample belongs to the drowsy or fresh class. Each model contributes one vote, and the ultimate prediction, denoted as HBPred, is determined by the majority of votes from these models for the drowsy or fresh class.
**Algorithm 1** RF-MLP Algorithm for Drowsiness Prediction**Require:** CSFE Features, TrainedRF, TrainedMLP**Ensure:** Drowsy, Fresh  1:$T_{RF}\leftarrow \mathrm{TrainedRF}$  2:$T_{MLP}\leftarrow \mathrm{TrainedMLP}$  3:**for** *i* in Dataset **do**  4:   $RFPrediction\leftarrow T_{RF}\left(i\right)$  5:   $MLPPrediction\leftarrow T_{MLP}\left(i\right)$  6:   HVPred[i]←argmax{RFPrediction,MLPPrediction}  7:**end for**  8:**Output:** Drowsy | Fresh ←HVPred

The Algorithm 2 outlines the steps used by the RF-XGB-SVM ensemble model following the hard voting criteria. The trained Random Forest (TRF), Trained XGB (TXGB), and trained SVM (TSVM) models operate on the feature vector to predict whether a given sample belongs to the drowsy or fresh class. Each model contributes one vote, and the ultimate prediction, denoted as HVPred, is determined by the majority of votes from these models for the drowsy or fresh class.
**Algorithm 2** RF-SGB-SVM Algorithm for Drowsiness Prediction**Require:** CSFE Features, TrainedRF, TrainedXGB, TrainedSVM**Ensure:** Predictions (Drowsy or Fresh)  1:$T_{RF}\leftarrow \mathrm{TrainedRF}$  2:$T_{XGB}\leftarrow \mathrm{TrainedXGB}$  3:$T_{SVM}\leftarrow \mathrm{TrainedSVM}$  4:**for** each *i* in Dataset **do**  5:   $RFPrediction\leftarrow T_{RF}\left(i\right)$  6:   $XGBPrediction\leftarrow T_{XGB}\left(i\right)$  7:   $SVMPrediction\leftarrow T_{SVM}\left(i\right)$  8:   HVPred[i]←argmax({RFPrediction,XGBPrediction,SVMPrediction})  9:**end for**10:**Output:** Drowsy | Fresh ←HVPred

## 4. Results and Discussion

This section provides a comprehensive analysis and discussion of the results obtained from the experiments carried out during this research. The objective is to provide a thorough analysis of the results while clarifying their importance within the context of this study. Furthermore, it involves a substantial discussion exploring the impacts and importance of these findings, thereby enriching the understanding of the broader academic and practical implications stemming from the research endeavor.

### 4.1. Experiment Setup

The experimental analyses were conducted on the HP EliteBook x360 1040 G6 (HP Inc., Lahore, Pakistan), which serves as the primary computing platform. Equipped with an Intel(R) Core (TM) i5-8365U processor operating at 1.60 GHz, this system exhibits remarkable computational prowess at a peak speed of 1.90 GHz. An additional 16.0 GB of RAM significantly enhances the performance of the CPU, resulting in improved efficiency when it comes to multitasking and data management. Implementing Windows 11 Pro and running on a 64-bit architecture, the system demonstrates the seamless integration of state-of-the-art hardware and software components. This technical configuration highlights the utilization of advanced capabilities throughout the experimentation phase, ensuring a stable and flexible computing environment. Data preprocessing was performed using MATLAB R2020a. The subsequent experiments, including feature extraction and model training, were implemented in Python 3.0 using Jupyter Notebook 6.5.2. This environment allowed for the seamless integration of code, visualizations, and documentation, facilitating an interactive and iterative workflow. The software environment comprised Python 3.8, TensorFlow 2.4, and scikit-learn 0.24. Hyperparameter tuning was performed using grid search to identify optimal configurations for each model. Software debugging and iterative refinements were managed using Jupyter Notebook’s real-time monitoring and visualization tools, which allowed for dynamic adjustments during the training process.

### 4.2. Data Splitting

The dataset comprises recordings obtained from forty male participants, encompassing both drowsy and alert states. By segmenting each file at one-minute intervals, the total number of files within each category rises to 200. The dataset is subsequently divided into test and training sets in the proportion of 70% for training and 30% for testing. Additionally, a GAN is employed to augment the dataset, resulting in each class having 1200 values. These augmented datasets are then divided into training and testing sets in an 80–20 split, ensuring a robust and comprehensive evaluation of the model’s performance. The objective of this strategic division is to guarantee an equitable distribution of instances for drowsy and non-drowsy states throughout the training and testing stages, thereby promoting the development and assessment of resilient models.

### 4.3. Classification Results

In this study, a diverse array of machine learning classifiers, encompassing SVM, Random Forest (RF), XGBoost (XGB), and Multi-Layer Perceptron (MLP), were employed for the classification task. Furthermore, ensemble classifiers were implemented in two ways RF-MLP Ensemble and RF-XGB-SVM Ensemble. To improve the performance of the models, rigorous hyperparameter tuning was performed using the Grid Search technique. The selected specific hyperparameters are provided in Table 3.

The training phase included the use of the training dataset, followed by testing on an independent test set. Table 4 completely presents the classification performance of these models on the test set, providing insights into their efficacy and comparative evaluation.

**Figure 8:**
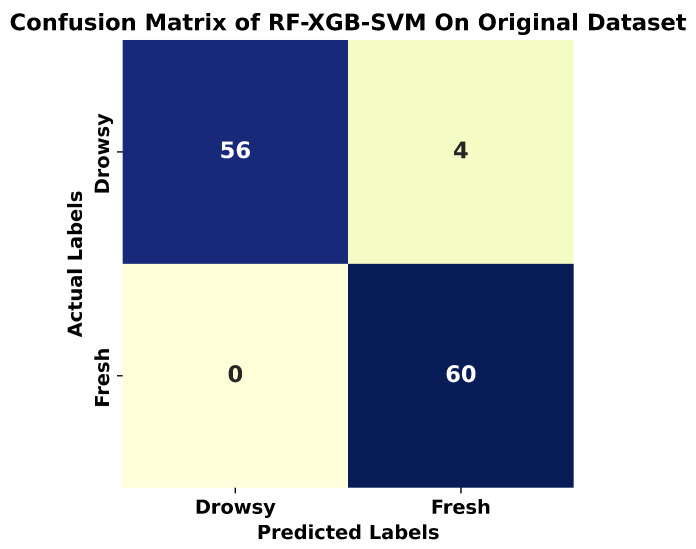
Confusion matrix of RF-XGB-SVM on original dataset.

The results in Table 4 show that ensemble models, RF-MLP and RF-XGB-SVM, showcased exceptional performance, with an accuracy of 95% and 96.6%, respectively. This strong result emphasizes the efficacy of combining several learning techniques, demonstrating their capacity to greatly improve predictive accuracy. Notably, RF consistently outperformed all the measures tested, attaining an amazing accuracy of 93.33% and an F1-score of 0.94. SVM and XGBoost performed similarly, with both models achieving a roughly 91% accuracy and F1 scores of 0.91 and 0.92, respectively. Although effective, they marginally trailed RF’s superior performance. The MLP performed significantly worse, with an accuracy of 74.6% and an F1-score of 0.75. This result sheds light on the potential limits of the MLP model architecture for the specific drowsiness detection task. For accurate drowsiness detection, the ensemble model, notably RF-XGB-SVM, emerges as a highly promising classifier. The confusion matrix is shown in Figure 8.

The augmented dataset was used to ensure fair and comparable evaluations across different models and datasets by maintaining consistency in model training. The same set of hyperparameters as those applied to the original dataset was used, guaranteeing uniform training conditions. Following successful training, the trained models were rigorously tested using the designated test set. The evaluation results, meticulously documented and presented in Table 5, provide insights into the classifiers’ performance metrics, including accuracy, precision, recall, and F1-score.

**Figure 9:**
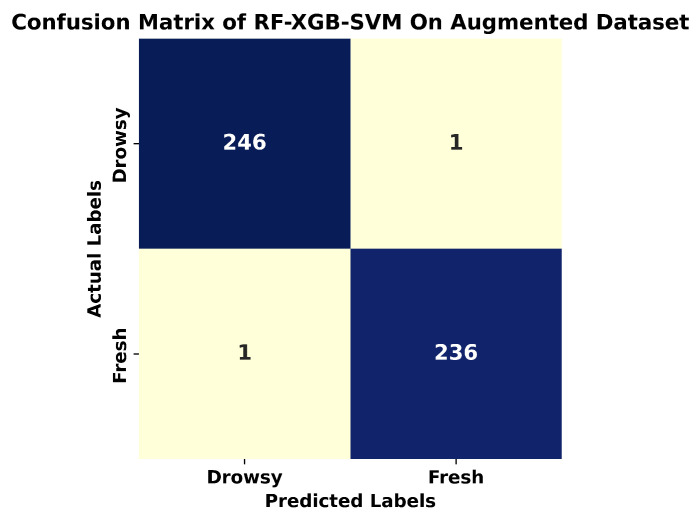
Confusion matrix of RF-XGB-SVM on augmented dataset.

It is evident from Table 5 that the SVM achieved an accuracy of 98.76%, with a Precision, Recall, and F1-Score all standing at 0.99, indicating a highly consistent and reliable performance across different evaluation metrics. The RF and XGB classifiers both exhibited identical performance metrics, each attaining an accuracy of 99.17%, and scoring 0.99 in Precision, Recall, and F1-Score. This suggests that both classifiers were equally effective in handling the augmented dataset, offering robust and accurate predictions. The MLP demonstrated the highest performance among the individual classifiers, with an accuracy of 99.5%. Remarkably, it achieved perfect scores of 100 in Precision, Recall, and F1-Score, indicating an exceptional ability to correctly identify and classify instances without any false positives or negatives. For the ensemble classifiers, the RF-MLP Ensemble achieved an accuracy of 99.3%, with a Precision, Recall, and F1-Score of 0.99. This performance is slightly lower than that of the MLP alone but still indicates strong predictive capabilities by leveraging the strengths of both RF and MLP. The RF-XGB-SVM Ensemble outperformed all other models, reaching an accuracy of 99.58%. It also achieved perfect scores of 100 in Precision, Recall, and F1-Score. This superior performance highlights the effectiveness of combining multiple classifiers, capitalizing on their individual strengths to deliver highly accurate and reliable predictions. The results demonstrate that all classifiers performed exceptionally well on the augmented dataset, with ensemble methods, particularly the RF-XGB-SVM Ensemble, providing a slightly higher accuracy than other classifiers. The confusion matrix of RF-XGB-SVM is shown in Figure 9.

### 4.4. K-fold Cross Validation

To assess the robustness and reliability of the models, a K-fold cross-validation approach was implemented in this study. The dataset underwent a process of partitioning into five distinct folds, and the models underwent iterative training and evaluation across each of these folds. Table 6 provides a comprehensive presentation of the results obtained from the cross-validation process. This enables readers to gain a nuanced comprehension of the performance of the models across various subsets of the data.

The findings presented in Table 4 indicate that the ensemble models, specifically RF-XGB-SVM, demonstrated superior performance in terms of both accuracy and consistency. It is noteworthy that RF-XGB-SVM demonstrated the highest accuracy at 97% and a remarkably low standard deviation of 0.018. These results indicate that RF-XGB-SVM operates with robustness and dependability across various factors. Indicating its effective generalization capabilities, RF-MLP additionally exhibited notable results, possessing a precision rate of 95% and a standard deviation of 0.03. In comparison to other individual classifiers, RF demonstrated its efficacy as a solitary model by attaining an accuracy of 94% and a moderate standard deviation of 0.02. In contrast, SVM and XGBoost exhibited similar performance levels, attaining accuracies of approximately 91% each. With a standard deviation of 0.04 compared to SVM’s 0.05, XGBoost exhibited marginally less variability. The MLP demonstrated the most substantial standard deviation of 0.04 and the lowest accuracy of 73%.

The k-fold cross-validation results on the augmented dataset, presented in Table 7, provide a comprehensive evaluation of the classifiers’ performance in terms of accuracy and variability. The accuracy is reported along with the standard deviation (Std), which indicates the consistency of the model across different folds. The SVM and RF classifiers both achieved an average accuracy of 0.98 with a standard deviation of 0.01. This reflects their robust performance and reliability, with minimal variation in accuracy across the different folds of the dataset. XGB exhibited a slightly lower average accuracy of 0.97 with a standard deviation of 0.01. While still demonstrating strong performance, the XGB classifier showed a slightly higher variability in its predictions compared to SVM and RF. The MLP classifier outperformed the other individual classifiers, achieving an impressive average accuracy of 0.99 with a standard deviation of 0.01. This high accuracy, coupled with low variability, underscores MLP’s effectiveness and stability in handling the augmented dataset. The RF-MLP Ensemble, which combines the strengths of both Random Forest and Multi-Layer Perceptron, achieved an average accuracy of 0.98 with a standard deviation of 0.01. This indicates that the ensemble method is as reliable as the individual RF and SVM classifiers, but did not surpass the performance of MLP alone. The RF-XGB-SVM Ensemble demonstrated the highest performance among all models, with an average accuracy of 0.99 and a standard deviation of 0.01. This suggests that combining Random Forest, XGBoost, and SVM in an ensemble approach results in a model that is not only highly accurate but also consistently reliable across different subsets of the dataset. The k-fold cross-validation results affirm the high performance and robustness of the classifiers, with ensemble methods, particularly the RF-XGB-SVM Ensemble, providing the best accuracy and consistency.

### 4.5. Computational Time Complexity

Table 8 summarizes the computational time complexity of classifiers, measured in seconds. SVM demonstrates the lowest time complexity at 1.53 s, followed by RF (2.47) and XGB (2.81). MLP has a higher complexity at 3.63 s, while the RF-MLP ensemble increases to 3.72 s. The RF-XGB-SVM ensemble requires the most time at 4.15 s. This highlights a trade-off between computational efficiency and model complexity, with simpler models offering faster predictions, while more complex ensembles deliver heightened accuracy at the expense of increased computational time.

The computational time complexity of the classifiers on the augmented dataset, as shown in Table 9, provides insight into the efficiency of each model in terms of training time measured in seconds. The SVM required 4.19 s for training, indicating a relatively fast processing time given its sophisticated algorithm. Similarly, the RF classifier took 4.22 s, which is comparable to SVM and reflects its efficiency in handling the dataset with multiple decision trees. XGB demonstrated the shortest computational time among all classifiers, completing its training in 3.93 s. This rapid processing time is indicative of XGB’s optimized implementation for gradient boosting, which is known for its speed and performance. The MLP, however, required the longest training time of 5.1 s. This increased time complexity can be attributed to the neural network’s iterative training process, involving numerous parameters and layers that need to be optimized. Among the ensemble classifiers, the RF-MLP Ensemble took 4.3 s to train. This slight increase compared to the individual RF model reflects the added complexity of integrating the MLP component, yet it remains efficient. The RF-XGB-SVM Ensemble had a computational time of 4.7 s. While this is higher than the individual classifiers, it remains reasonable given that it combines three different models. The increase in computational time is justified by the significant boost in accuracy and robustness provided by this ensemble approach. The computational time complexity results illustrate a trade-off between training time and model performance. While MLP and ensemble methods take longer to train, their superior accuracy and reliability often justify the additional computational cost. Conversely, XGB stands out for its quick processing time, making it an efficient choice when computational resources or time are limited.

### 4.6. Comparison with Existing Studies

In comparison to a prior study conducted by Siddiqui et al. [12], which used the same dataset as employed in this manuscript, the proposed method presented in this manuscript has exhibited advancements in accuracy as shown in Table 10. The study [12] achieved an accuracy of 87.5%, while our proposed methodology achieved a significantly higher accuracy of 99.58%. This substantial improvement underscores the efficacy of the approach introduced in this manuscript. The enhanced accuracy suggests that the employed classifiers, such as RF-XGB-SVM, have effectively leveraged the features within the dataset, surpassing the performance achieved in the earlier study.

### 4.7. Discussion

The results demonstrate that the RF-XGB-SVM ensemble model outperforms all other classifiers. In multiple evaluation criteria, such as accuracy, precision, recall, and F1-score, RF-XGB-SVM consistently exhibited superior performance compared to its competitors. The remarkable efficacy of the RF-XGB-SVM ensemble model can be attributed to the synergistic cooperation of RF, XGB, and SVM. RF, with its collection of decision trees, effectively captures complex data relationships. The XGB algorithm, a robust gradient boosting technique, enhances the performance of less capable models, while SVM prioritizes the identification of suitable hyperplanes for classification. The integration of these classifiers produces a model that not only utilizes a range of learning techniques but also performs exceptionally well in detecting different patterns throughout the feature space. The ensemble approach offers a reliable solution by reducing overfitting and allowing error correction through the combined knowledge of classifiers for drowsiness detection. Despite its excellent classification performance, it is noteworthy that the RF-XGB-SVM model incurs a higher computational time compared to individual classifiers.

**Figure 10:**
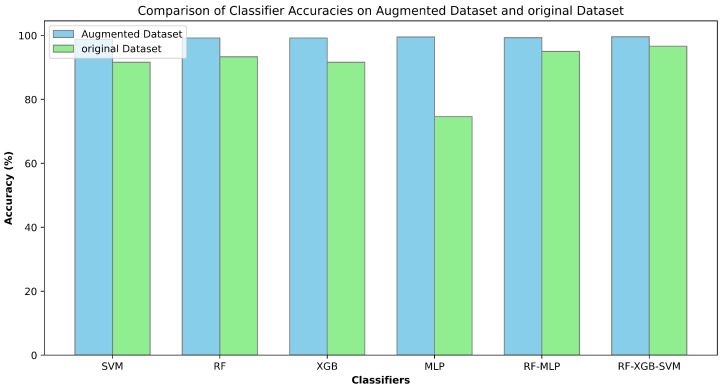
Comparison of accuracies on both datasets.

The accuracy comparison of all the classifiers on both datasets is shown in Figure 10. The analysis revealed that while individual models like SVM, RF, XGBoost, and MLP performed exceptionally well, achieving high accuracy rates (up to 99.5% for MLP), ensemble methods provided the best results. The RF-XGB-SVM ensemble achieved the highest accuracy of 99.58%, coupled with perfect precision, recall, and F1-score, demonstrating the advantage of combining diverse classifiers. K-fold cross-validation confirmed the robustness and consistency of all models, with low standard deviations indicating reliable performance across different folds.

The findings highlight the effectiveness of ensemble approaches in achieving high performance while balancing computational efficiency. The primary aim of this study is to achieve high accuracy in detecting driver drowsiness, which is crucial for enhancing road safety. This focus, however, leads to higher computational complexity. The benefits of improved detection accuracy justify the additional computational cost. To make the method more practical for deployment in various real-world scenarios, efforts are being made to explore optimizations that improve real-time performance.

## 5. Conclusions

Drowsiness while driving offers a significant risk, resulting in decreased cognitive performance and an increased likelihood of an accident. Drowsiness-related vehicle crashes have serious consequences, including trauma, economic costs, injuries, and even fatalities. This study demonstrates the effectiveness of using UWB radar and advanced ensemble models for real-time driver drowsiness detection. This study focuses on classifying drivers into drowsy and non-drowsy states using data from ultra-wideband radar. The five-minute dataset was divided into one-minute chunks and converted to grayscale images. A Two-Dimensional Convolutional Neural Network was used to extract spatial features from these images. Using these features, various machine learning classifiers were trained and tested. Notably, the ensemble classifier RF-XGB-SVM attained an amazing accuracy of 96.6% by combining Random Forest, XGBoost, and Support Vector Machine. The k-fold cross-validation score was 97%, with a standard deviation of 0.018, indicating a stable and consistent performance. Utilizing Generative Adversarial Networks for dataset augmentation led to enhanced accuracies across all models, with the RF-XGB-SVM model surpassing others by achieving an accuracy score of 99.58%. The proposed method significantly improves detection accuracy, highlighting its potential to enhance road safety by reducing fatigue-related accidents. Future research could investigate the integration of other sensor modalities for improved detection, as well as the deployment of the system in real-world driving scenarios for comprehensive validation.

## Figures and Tables

**Table 1:** Neural Network Model Configuration.

Layer Type	Configuration
Input Rescaling	Scaling factor: 1.0/255
Convolutional (Conv2D)	Filters: 64, Kernel: (3, 3), Activation: ReLU
Max Pooling (MaxPooling2D)	Pool Size: (2, 2)
Convolutional (Conv2D)	Filters: 32, Kernel: (3, 3), Activation: ReLU
Max Pooling (MaxPooling2D)	Pool Size: (2, 2)
Flatten	N/A
Dense	Neurons: 128, Activation: ReLU

**Table 2:** Snippets of the dataset post augmentation.

Sr. No.	0	1	2	⋯	97	98	99	Label
0	0.39622176	0.4066065	0.51660347	⋯	0.46284893	0.48666704	0.3540284	0
1	0.41745254	0.54292786	0.5974161	⋯	0.4108211	0.47298706	0.3946942	0
2	0.4305402	0.48049223	0.5213418	⋯	0.40519458	0.6011478	0.22075108	0
3	0.422287	0.6520922	0.63822633	⋯	0.34400716	0.442513	0.39881736	0
4	0.41309932	0.47723842	0.51149786	⋯	0.3870272	0.5330019	0.20417304	0
5	0.52232856	0.45020208	0.57795453	⋯	0.46976835	0.4652797	0.17163844	0
6	0.4189465	0.47918236	0.51208353	⋯	0.3875902	0.5244716	0.19999994	0
7	0.40711606	0.33762354	0.5082019	⋯	0.5300679	0.5099058	0.32978377	0
8	0.43408674	0.6784697	0.64829403	⋯	0.33602956	0.43760943	0.38681032	0
…	⋯	⋯	⋯	⋯	⋯	⋯	⋯	⋯
2391	0.5387267	0.540245	0.6575716	⋯	0.4398443	0.5939529	0.30980185	1
2392	0.5398885	0.62441266	0.6159498	⋯	0.39132738	0.79611707	0.20297673	1
2393	0.5654393	0.59164715	0.63794005	⋯	0.4651416	0.7560228	0.23912714	1
2394	0.5286886	0.5439412	0.6900763	⋯	0.46690488	0.5235571	0.33242399	1
2395	0.34617162	0.47954148	0.5320659	⋯	0.49993005	0.40980458	0.19199125	1
2396	0.53729063	0.5442955	0.67633796	⋯	0.4568716	0.55147946	0.32810143	1
2397	0.5563764	0.61615664	0.633111	⋯	0.4239145	0.7834681	0.22323802	1
2398	0.5673374	0.5729127	0.6460825	⋯	0.47191876	0.7099173	0.26245716	1
2399	0.49523562	0.5511889	0.705765	⋯	0.47301993	0.4585747	0.34522685	1

**Table 3:** Classifiers and Their Hyperparameters.

Classifiers	Hyperparameters
SVM	C = 10, kernel = ‘rbf’
RF	max_depth = None, n_estimators = 100
XGB	learning_rate = 0.2, n_estimators = 50
MLP	alpha = 0.001, hidden_layer_sizes = (100,)
RF-MLP	RF (max_depth = None, n_estimators = 100), MLP (alpha = 0.001, hidden_layer_sizes = (100,))
RF-XGB-SVM	RF (max_depth = None, n_estimators = 100), XGB (learning_rate = 0.2, n_estimators = 50), SVM (C = 10, kernel = ‘rbf’)

**Table 4:** Classification matrices of the classifiers on the test data.

Classifiers	Accuracy (%)	Precision	Recall	F1-Score
SVM	91.6	0.91	0.91	0.91
RF	93.33	0.94	0.94	0.94
XGB	91.6	0.91	0.92	0.92
MLP	74.6	0.74	0.75	0.75
RF-MLP	95	0.95	0.95	0.95
RF-XGB-SVM	96.6	0.97	0.97	0.97

**Table 5:** Results of classifier on Augmented dataset.

Classifiers	Accuracy (%)	Precision	Recall	F1-Score
SVM	98.76	0.99	0.99	0.99
RF	99.17	0.99	0.99	0.99
XGB	99.17	0.99	0.99	0.99
MLP	99.5	100	100	100
RF-MLP	99.3	0.99	0.99	0.99
RF-XGB-SVM	99.58	100	100	100

**Table 6:** Kfold cross-validation results on original dataset.

Classifiers	Accuracy ± Std
SVM	0.91 ± 0.05
RF	0.94 ± 0.02
XGB	0.91 ± 0.04
MLP	0.73 ± 0.04
RF-MLP	0.95 ± 0.03
RF-XGB-SVM	0.97 ± 0.018

**Table 7:** Kfold cross-validation results on augmented dataset.

Classifiers	Accuracy ± Std
SVM	0.98 ± 0.01
RF	0.98 ± 0.01
XGB	0.97 ± 0.01
MLP	0.99 ± 0.01
RF-MLP	0.98 ± 0.01
RF-XGB-SVM	0.99 ± 0.01

**Table 8:** Computational time complexity of classifiers.

Classifiers	Computational Time (s)
SVM	1.53
RF	2.47
XGB	2.81
MLP	3.63
RF-MLP	3.72
RF-XGB-SVM	4.15

**Table 9:** Computational time complexity of classifiers on Augmented dataset.

Classifiers	Computational Time (s)
SVM	4.19
RF	4.22
XGB	3.93
MLP	5.1
RF-MLP	4.3
RF-XGB-SVM	4.7

**Table 10:** Comparison with other Studies.

Study	Accuracy
[12]	87.5%
Proposed	99.58%

## Data Availability

This research has no associated data.

## References

[1] Martiniuk A.L., Senserrick T., Lo S., Williamson A., Du W., Grunstein R.R., Woodward M., Glozier N., Stevenson M., Norton R. (2013). Sleep-deprived young drivers and the risk for crash: The DRIVE prospective cohort study. JAMA Pediatr..

[2] World Health Organization (2015). Global Status Report on Road Safety 2015.

[3] Council N.S. Drivers Are Falling Asleep behind the Wheel. https://www.nsc.org/road/safety-topics/fatigued-driver?.

[4] Drowsy Driving and Automobile Crashes. https://www.nhtsa.gov/sites/nhtsa.gov/files/808707.pdf.

[5] Chand H.V., Karthikeyan J. (2022). CNN Based Driver Drowsiness Detection System Using Emotion Analysis. Intell. Autom. Soft Comput..

[6] Fouad I.A. (2023). A robust and efficient EEG-based drowsiness detection system using different machine learning algorithms. Ain Shams Eng. J..

[7] Jan M.T., Hashemi A., Jang J., Yang K., Zhai J., Newman D., Tappen R., Furht B. (2022). Non-intrusive drowsiness detection techniques and their application in detecting early dementia in older drivers. Future Technologies Conference.

[8] Magán E., Sesmero M.P., Alonso-Weber J.M., Sanchis A. (2022). Driver drowsiness detection by applying deep learning techniques to sequences of images. Appl. Sci..

[9] Nasri I., Karrouchi M., Kassmi K., Messaoudi A. (2022). A Review of Driver Drowsiness Detection Systems: Techniques, Advantages and Limitations. arXiv.

[10] Rajkar A., Kulkarni N., Raut A. (2022). Driver drowsiness detection using deep learning. Applied Information Processing Systems: Proceedings of ICCET 2021.

[11] Saleem A.A., Siddiqui H.U.R., Raza M.A., Rustam F., Dudley S., Ashraf I. (2023). A systematic review of physiological signals based driver drowsiness detection systems. Cogn. Neurodyn..

[12] Siddiqui H.U.R., Saleem A.A., Brown R., Bademci B., Lee E., Rustam F., Dudley S. (2021). Non-invasive driver drowsiness detection system. Sensors.

[13] Thota J.R., Jaidhan B., Jitendra M.S., Shanmuk Srinivas A., Venkata Praneel A. (2022). Computer Vision-Based Alert System to Detect Fatigue in Vehicle Drivers. Advances in Data Science and Management: Proceedings of ICDSM 2021.

[14] Zilberg E., Burton D., Xu M., Karrar M., Lal S. (2022). Methodology and initial analysis results for development of non-invasive and hybrid driver drowsiness detection systems. Advances in Broadband Communication and Networks.

[15] Albadawi Y., Takruri M., Awad M. (2022). A review of recent developments in driver drowsiness detection systems. Sensors.

[16] Sahayadhas A., Sundaraj K., Murugappan M. (2012). Detecting driver drowsiness based on sensors: A review. Sensors.

[17] Triyanti V., Iridiastadi H. (2017). Challenges in detecting drowsiness based on driver’s behavior. IOP Conference Series: Materials Science and Engineering.

[18] Budak U., Bajaj V., Akbulut Y., Atila O., Sengur A. (2019). An effective hybrid model for EEG-based drowsiness detection. IEEE Sens. J..

[19] Cui J., Lan Z., Sourina O., Müller-Wittig W. (2022). EEG-based cross-subject driver drowsiness recognition with an interpretable convolutional neural network. IEEE Trans. Neural Netw. Learn. Syst..

[20] Jiang Y., Zhang Y., Lin C., Wu D., Lin C.T. (2020). EEG-based driver drowsiness estimation using an online multi-view and transfer TSK fuzzy system. IEEE Trans. Intell. Transp. Syst..

[21] Mardi Z., Ashtiani S.N.M., Mikaili M. (2011). EEG-based drowsiness detection for safe driving using chaotic features and statistical tests. J. Med. Signals Sens..

[22] Noori S.M.R., Mikaeili M. (2016). Driving drowsiness detection using fusion of electroencephalography, electrooculography, and driving quality signals. J. Med. Signals Sens..

[23] Ren Z., Li R., Chen B., Zhang H., Ma Y., Wang C., Lin Y., Zhang Y. (2021). EEG-based driving fatigue detection using a two-level learning hierarchy radial basis function. Front. Neurorobot..

[24] Tuncer T., Dogan S., Subasi A. (2021). EEG-based driving fatigue detection using multilevel feature extraction and iterative hybrid feature selection. Biomed. Signal Process. Control.

[25] Barua S., Ahmed M.U., Ahlström C., Begum S. (2019). Automatic driver sleepiness detection using EEG, EOG and contextual information. Expert Syst. Appl..

[26] Chieh T.C., Mustafa M.M., Hussain A., Hendi S.F., Majlis B.Y. (2005). Development of vehicle driver drowsiness detection system using electrooculogram (EOG). Proceedings of the 2005 1st International Conference on Computers, Communications, & Signal Processing with Special Track on Biomedical Engineering.

[27] Hayawi A.A., Waleed J. (2019). Driver’s drowsiness monitoring and alarming auto-system based on EOG signals. Proceedings of the 2019 2nd International Conference on Engineering Technology and Its Applications (IICETA).

[28] Jiao Y., Deng Y., Luo Y., Lu B.L. (2020). Driver sleepiness detection from EEG and EOG signals using GAN and LSTM networks. Neurocomputing.

[29] Wang H., Wu C., Li T., He Y., Chen P., Bezerianos A. (2019). Driving fatigue classification based on fusion entropy analysis combining EOG and EEG. IEEE Access.

[30] Zhu X., Zheng W.L., Lu B.L., Chen X., Chen S., Wang C. (2014). EOG-based drowsiness detection using convolutional neural networks. Proceedings of the 2014 International Joint Conference on Neural Networks (IJCNN).

[31] Ebrahimian S., Nahvi A., Tashakori M., Salmanzadeh H., Mohseni O., Leppänen T. (2022). Multi-Level Classification of Driver Drowsiness by Simultaneous Analysis of ECG and Respiration Signals Using Deep Neural Networks. Int. J. Environ. Res. Public Health.

[32] Kiashari S.E.H., Nahvi A., Bakhoda H., Homayounfard A., Tashakori M. (2020). Evaluation of driver drowsiness using respiration analysis by thermal imaging on a driving simulator. Multimed. Tools Appl..

[33] Lee B.G., Lee B.L., Chung W.Y. (2014). Mobile healthcare for automatic driving sleep-onset detection using wavelet-based EEG and respiration signals. Sensors.

[34] Musicant O., Richmond-Hacham B., Botzer A. Estimating Driver Fatigue Based on Heart Activity, Respiration Rate. Proceedings of the Lindholmen Conference Centre.

[35] Solaz J., Laparra-Hernández J., Bande D., Rodríguez N., Veleff S., Gerpe J., Medina E. (2016). Drowsiness detection based on the analysis of breathing rate obtained from real-time image recognition. Transp. Res. Procedia.

[36] Arefnezhad S., Eichberger A., Frühwirth M., Kaufmann C., Moser M. (2020). Driver drowsiness classification using data fusion of vehicle-based measures and ECG signals. Proceedings of the 2020 IEEE International Conference on Systems, Man, and Cybernetics (SMC).

[37] Babaeian M., Mozumdar M. (2019). Driver drowsiness detection algorithms using electrocardiogram data analysis. Proceedings of the 2019 IEEE 9th Annual Computing and Communication Workshop and Conference (CCWC).

[38] Yaacob S., Affandi N.A.I., Krishnan P., Rasyadan A., Yaakop M., Mohamed F. (2020). Drowsiness detection using EEG and ECG signals. Proceedings of the 2020 IEEE 2nd International Conference on Artificial Intelligence in Engineering and Technology (IICAIET).

[39] Fan Y., Gu F., Wang J., Wang J., Lu K., Niu J. (2021). SafeDriving: An effective abnormal driving behavior detection system based on EMG signals. IEEE Internet Things J..

[40] Naim F., Mustafa M., Sulaiman N., Rahman N.A.A. (2022). The study of time domain features of EMG signals for detecting driver’s drowsiness. Recent Trends in Mechatronics Towards Industry 4.0: Selected Articles from iM3F 2020, Malaysia.

[41] Rahman N.A., Mustafa M., Sulaiman N., Samad R., Abdullah N. (2022). EMG signal segmentation to predict driver’s vigilance state. Human-Centered Technology for a Better Tomorrow: Proceedings of HUMENS 2021.

[42] Satti A.T., Kim J., Yi E., Cho H.Y., Cho S. (2021). Microneedle array electrode-based wearable EMG system for detection of driver drowsiness through steering wheel grip. Sensors.

[43] Wali M.K. (2020). Ffbpnn-based high drowsiness classification using EMG and WPT. Biomed. Eng. Appl. Basis Commun..

[44] Xie A. (2012). Effect of sleep on breathing-why recurrent apneas are only seen during sleep. J. Thorac. Dis..

[45] Warwick B., Symons N., Chen X., Xiong K. (2015). Detecting driver drowsiness using wireless wearables. Proceedings of the 2015 IEEE 12th International Conference on Mobile Ad Hoc and Sensor Systems.

[46] Yang C., Wang X., Mao S. (2020). Respiration monitoring with RFID in driving environments. IEEE J. Sel. Areas Commun..

[47] Brown R., Ghavami N., Adjrad M., Ghavami M., Dudley S. (2017). Occupancy based household energy disaggregation using ultra wideband radar and electrical signature profiles. Energy Build..

[48] Chong C.C., Watanabe F., Inamura H. (2006). Potential of UWB technology for the next generation wireless communications. Proceedings of the 2006 IEEE Ninth International Symposium on Spread Spectrum Techniques and Applications.

[49] Tsang T.K., El-Gamal M.N. (2005). Ultra-wideband (UWB) communications systems: An overview. Proceedings of the 3rd International IEEE-NEWCAS Conference.

[50] Wang X., Dinh A., Teng D. (2012). Radar sensing using ultra wideband–design and implementation. Ultra Wideband—Current Status and Future Trends.

[51] Rana S.P., Dey M., Siddiqui H.U., Tiberi G., Ghavami M., Dudley S. (2017). UWB localization employing supervised learning method. Proceedings of the 2017 IEEE 17th International Conference on Ubiquitous Wireless Broadband (ICUWB).

[52] Rana S.P., Dey M., Brown R., Siddiqui H.U., Dudley S. Remote vital sign recognition through machine learning augmented UWB. Proceedings of the 12th European Conference on Antennas and Propagation (EuCAP 2018).

[53] Zafar K., Siddiqui H.U.R., Majid A., Saleem A.A., Raza A., Rustam F., Dudley S. (2023). Deep Learning Based Feature Engineering to Detect Anterior and Inferior Myocardial Infarction using UWB Radar Data. IEEE Access.

[54] Gao Z., Wang X., Yang Y., Mu C., Cai Q., Dang W., Zuo S. (2019). EEG-based spatio–temporal convolutional neural network for driver fatigue evaluation. IEEE Trans. Neural Netw. Learn. Syst..

[55] Pandey N.N., Muppalaneni N.B. (2021). Temporal and spatial feature based approaches in drowsiness detection using deep learning technique. J. Real-Time Image Process..

[56] Babaeian M., Amal Francis K., Dajani K., Mozumdar M. (2019). Real-time driver drowsiness detection using wavelet transform and ensemble logistic regression. Int. J. Intell. Transp. Syst. Res..

[57] Awais M., Badruddin N., Drieberg M. (2017). A hybrid approach to detect driver drowsiness utilizing physiological signals to improve system performance and wearability. Sensors.

[58] Suresh A., Naik A.S., Pramod A., Kumar N.A., Mayadevi N. (2023). Analysis and Implementation of Deep Convolutional Neural Network Models for Intelligent Driver Drowsiness Detection System. Proceedings of the 2023 7th International Conference on Intelligent Computing and Control Systems (ICICCS).

[59] Li A., Ma X., Guo J., Zhang J., Wang J., Zhao K., Li Y. (2023). Driver fatigue detection and human-machine cooperative decision-making for road scenarios. Multimed. Tools Appl..

[60] Majeed F., Shafique U., Safran M., Alfarhood S., Ashraf I. (2023). Detection of drowsiness among drivers using novel deep convolutional neural network model. Sensors.

[61] Shakeel M.F., Bajwa N.A., Anwaar A.M., Sohail A., Khan A. (2019). Detecting driver drowsiness in real time through deep learning based object detection. International Work-Conference on Artificial Neural Networks.

[62] Mohan R., Chalasani S., Mary S.S.C., Chauhan A., Parte S.A., Anusuya S. (2023). Identification of Driver Drowsiness Detection using a Regularized Extreme Learning Machine. Proceedings of the 2023 Second International Conference on Electronics and Renewable Systems (ICEARS).

[63] Miah A.A., Ahmad M., Mim K.Z. (2020). Drowsiness detection using eye-blink pattern and mean eye landmarks’ distance. Proceedings of the International Joint Conference on Computational Intelligence: IJCCI 201.

[64] Kawtikwar V.N., Tiwari G., Patil C., Pandey N., Tiwari P. (2023). Eyes on the Road: A Machine Learning-based Fatigue Detection System for Safer Driving. Proceedings of the 2023 International Conference on Inventive Computation Technologies (ICICT).

[65] Liu S., Zhao L., Yang X., Du Y., Li M., Zhu X., Dai Z. (2022). Remote drowsiness detection based on the mmwave fmcw radar. IEEE Sens. J..

[66] Ananthi S., Sathya R., Vaidehi K., Vijaya G. (2023). Drivers Drowsiness Detection using Image Processing and I-Ear Techniques. Proceedings of the 2023 7th International Conference on Intelligent Computing and Control Systems (ICICCS).

[67] Bajaj J.S., Kumar N., Kaushal R.K., Gururaj H., Flammini F., Natarajan R. (2023). System and method for driver drowsiness detection using behavioral and sensor-based physiological measures. Sensors.

[68] Srivastava A., Bansal S., Sehgal S.S. (2022). Real-Time Based Driver’s Drowsiness and Fatigue Detection System. Proceedings of the 2022 International Conference on Cyber Resilience (ICCR).

[69] Suresh Y., Khandelwal R., Nikitha M., Fayaz M., Soudhri V. (2021). Driver drowsiness detection using deep learning. Proceedings of the 2021 2nd International Conference on Smart Electronics and Communication (ICOSEC).

[70] Civik E., Yuzgec U. (2023). Real-time driver fatigue detection system with deep learning on a low-cost embedded system. Microprocess. Microsyst..

[71] Kannan R., Jahnavi P., Megha M. (2023). Driver Drowsiness Detection and Alert System. Proceedings of the 2023 IEEE International Conference on Integrated Circuits and Communication Systems (ICICACS).

[72] Kumar D., Nair S.R., Jayaraj R. (2023). Driver Drowsiness Detection Using Open CV and DLIB. Proceedings of the 2023 International Conference on Networking and Communications (ICNWC).

[73] Goodfellow I., Pouget-Abadie J., Mirza M., Xu B., Warde-Farley D., Ozair S., Courville A., Bengio Y. (2020). Generative adversarial networks. Commun. ACM.

